# Advancing Risk Assessment of Intermediate Risk Prostate Cancer Patients

**DOI:** 10.3390/cancers11060855

**Published:** 2019-06-20

**Authors:** Darrel Drachenberg, Julius A. Awe, Aline Rangel Pozzo, Jeff Saranchuk, Sabine Mai

**Affiliations:** 1 Section of Urology, Department of Surgery, Manitoba Prostate Center, Cancer Care Manitoba, University of Manitoba, Winnipeg, MB R3E 0V9, Canada; drach13@mymts.net (D.D.); jsaranchuk@exchange.hsc.mb.ca (J.S.); 2 Cell Biology, Research Institute of Hematology and Oncology, Cancer Care Manitoba, University of Manitoba, Winnipeg, MB R3E 0V9, Canada; d4mostcts@yahoo.com (J.A.A.); aline.rangelpozzo@umanitoba.ca (A.R.P.)

**Keywords:** intermediate risk prostate cancer, Gleason 7, circulating tumor cells, three-dimensional (3D) imaging, 3D nucleus, quantitative 3D telomere analysis

## Abstract

The individual risk to progression is unclear for intermediate risk prostate cancer patients. To assess their risk to progression, we examined the level of genomic instability in circulating tumor cells (CTCs) using quantitative three-dimensional (3D) telomere analysis. Data of CTCs from 65 treatment-naïve patients with biopsy-confirmed D’Amico-defined intermediate risk prostate cancer were compared to radical prostatectomy pathology results, which provided a clinical endpoint to the study and confirmed pre-operative pathology or demonstrated upgrading. Hierarchical centroid cluster analysis of 3D pre-operative CTC telomere profiling placed the patients into three subgroups with different potential risk of aggressive disease. Logistic regression modeling of the risk of progression estimated odds ratios with 95% confidence interval (CI) and separated patients into “stable” vs. “risk of aggressive” disease. The receiver operating characteristic (ROC) curve showed an area under the curve (AUC) of 0.77, while prostate specific antigen (PSA) (AUC of 0.59) and Gleason 3 + 4 = 7 vs. 4 + 3 = 7 (*p* > 0.6) were unable to predict progressive or stable disease. The data suggest that quantitative 3D telomere profiling of CTCs may be a potential tool for assessing a patient’s prostate cancer pre-treatment risk.

## 1. Introduction

Prostate cancer is the second most common cancer in men and is a heterogeneous disease with both indolent and more aggressive forms. Gleason patterns and Gleason scores have long been the single most important predictors of cancer treatment prognosis. However, patients with the same Gleason score 7 can often have vastly different outcomes [1,2,3,4,5]. With current methods, the clinical prognostic grouping for localized prostate cancer is imprecise, with 30–50% of patients recurring after image-guided radiotherapy or radical prostatectomy [1]. Close to 20% of intermediate-risk patients develop biochemical failure that occurs within 18 months of primary local therapy [1]. The consequence of imprecise clinical prognostic grouping is that some indolent tumors are overtreated, while more aggressive ones may mistakenly receive no or delayed treatment [6,7].

The apparent paradox in clinical prognostic grouping of patients and the inter- and intra-personal differences between patients of the same pathology grouping is linked to the level of genomic instability present in the patient’s tumor: More genomic instability in individual tumor cells sets the stage for ongoing tumor cell (micro)evolution and leads to the generation of several subsets of tumor cells with distinct genetic properties. These genetic variants contribute to tumor development and progression [1,2,3,4,5,8,9].

Since tissue biopsies rarely represent the whole tumor due to inherent sampling issues, liquid biopsies become an attractive option in the assessment of a patient’s tumor [10,11]. They offer the opportunity of multiple and less invasive sampling from the patient’s blood and allow for the monitoring of the dynamic process of tumor evolution (for review, see [12]). Liquid biopsies have commonly focused on the analysis of circulating tumor cells (CTCs), cell-free nuclei acids (DNA and RNA) as well as exosomes; among all of these types of liquid biopsies, most work has been done with CTCs. While their limitations are linked to their rare numbers or even absence in some patients, a difficulty that is also encountered by other types of liquid biopsies, the use of CTCs in the clinic is becoming a reality [10,13]. Clinical validation for CTCs in metastatic-castration-resistant prostate cancer is most mature [10,13,14,15]. One prominent example is the nuclear-specific androgen receptor variant 7 (AR-V7) detection on CTCs in this patient group [16]. The presence or absence of this protein is required to inform treatment decisions for these patients [16].

Our current study focused on intermediate risk prostate cancer. This risk group is defined as Gleason 3 + 4 = 7 or 4 + 3 = 7, a serum prostate-specific antigen (PSA) between 10–20 ng/mL and clinical T2b or T2c disease. The heterogeneity of this patient group is well described, and the risk prediction for an individual patient in this group needs improvement especially if one is to utilize active surveillance as a possible disease management approach for a subset of these patients [8]. 

To identify the level of genomic instability present in intermediate risk prostate cancer patients’ CTCs and to potentially link this genomic hallmark to the risk to disease progression, we applied three-dimensional (3D) fluorescent telomere imaging and profiling to CTCs. 3D telomere profiling is a quantitative assay [17]: it determines the level of ongoing genomic instability by measuring telomere-specific structural genetic parameters of each nucleus as previously reported [18,19,20,21]. This includes the measurement of the level of aneuploidy (gain and loss of chromosomes) present in each nucleus using the fluorescent telomere signal numbers as indicators [22,23]. 3D telomere profiling further determines the level of ongoing genomic rearrangements through the measurement of telomeric aggregates (clusters of telomeres) and telomere lengths [24]. Nuclear volumes are also determined and telomere numbers per nuclear volume assessed [17]. The single nucleus readout of 3D telomere measurements therefore is the immediate summary of the genomic instability found in every cell. Moreover, these measurements enable the determination of genomic cell-to-cell heterogeneity [25]. This technology has been previously applied to other cancers and was applied to subgroup patients [26,27,28,29,30,31].

Using a filtration-based technique (ScreenCell, Paris, France) [32], we have successfully isolated CTCs from different cancers including prostate adenocarcinoma [25,33]. We have also shown that this filtration device is suitable to isolate CTCs from all prostate cancer risk groups, including the intermediate risk group of prostate cancer [34].

In this current report, 3D telomere profiling was carried out in a blinded fashion, with 65 treatment-naïve intermediate risk prostate cancer patients consenting to having their blood drawn for the 3D telomere analysis of their CTCs. The study endpoint was the final pathology report after radical prostatectomy (RP). At that time, CTC telomere profiling data and pathology reports were compared, and statistical analyses performed. We report on the ability of 3D telomere profiling to identify patients with stable or aggressive/progressive forms of the disease. 

## 2. Results

### 2.1. Pathology Reports after Radical Prostatectomy Show Close Association with Pre-Operative 3D Profiling Results of CTCs

All patients included in our pilot study underwent radical prostatectomy (RP). RP pathology results were compared with the statistical analysis of the 3D telomere profiles obtained from pre-operative 3D telomere profiling of CTCs. Table 1 shows intermediate risk prostate cancer patients who, at RP, had the stable form of the disease when compared to pre-operative biopsy or had disease upgrading. The latter is highlighted in bold in Table 1. The RP reports of the latter patients indicated that the tumor was no longer Gleason 7 (3 + 4) but displayed a primary pattern 4 or > Gleason 7, which showed tertiary pattern 5 and/or lymph node involvement. 

### 2.2. Three-Dimensional (3D) Telomere Profiling of CTCs from Intermediate Risk Prostate Cancer Patients

**Figure 1:**
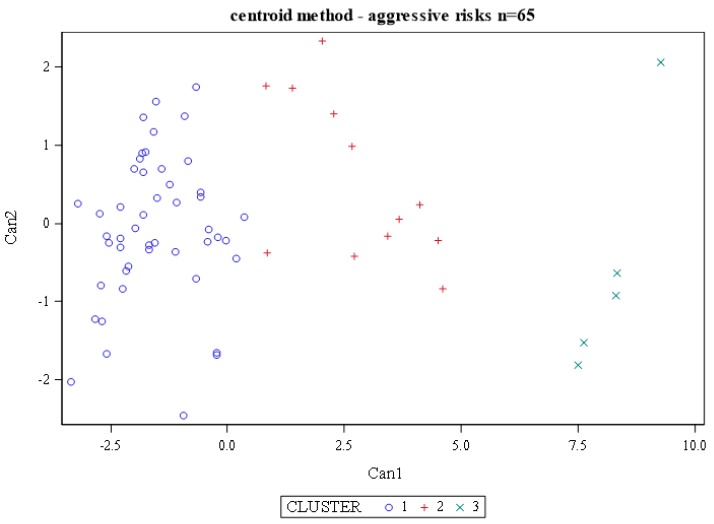
Centroid cluster analysis of 3D nuclear profiling of circulating tumor cells (CTCs) from 65 patients with intermediate risk prostate cancer. A combination of telomere parameters (Materials and Methods) led to the stratification of patients into clusters, each with a different level of genomic instability and a different risk to progression. Patients in cluster 3 (green) had the lowest risk (20%) to progression, while those in cluster two (red) and cluster 1 (blue) had an intermediate (50%) to high risk (68.75%), respectively.

Three-dimensional (3D) telomere analysis using TeloView^TM^ analysis [17] of CTCs from each of the patients provided data for each telomere in each nucleus. Hierarchical centroid cluster analysis was performed with all data sets, and the patients were grouped into three subgroups (clusters 1–3). Figure 1 shows the distribution of the patients after clustering.

The 3D telomere profiling of CTCs established a clear association with the RP pathology findings summarized in Table 1. The three clusters identified from 3D telomere profiling data after hierarchical centroid cluster analysis identified patients with different levels of genomic instability and different risk to progression (Figure 1). Cluster 3 contains predominantly stable intermediate risk prostate cancer patients, in which 20% of the patients have aggressive disease. This cluster has one outlier in the top right corner. This patient’s CTCs had comparatively larger nuclei and more telomeres per nuclear volume than the rest of cluster 3. However, overall, the other telomere parameters placed him in this cluster. In contrast, cluster 2 of intermediate risk prostate cancer includes an intermediate risk group, in which 50% of the patients represent those with aggressive disease. The higher risk grouping of the intermediate risk prostate cancer group is found in cluster 1 and comprises 68.75% of the patients in our pilot study. Thus, as patients are observed in clusters 3, 2 or 1, their disease appears more aggressive (cluster 1 > cluster 2 > cluster 3) based on their genomic instability pattern detected by 3D telomere analysis.

**Figure 2:**
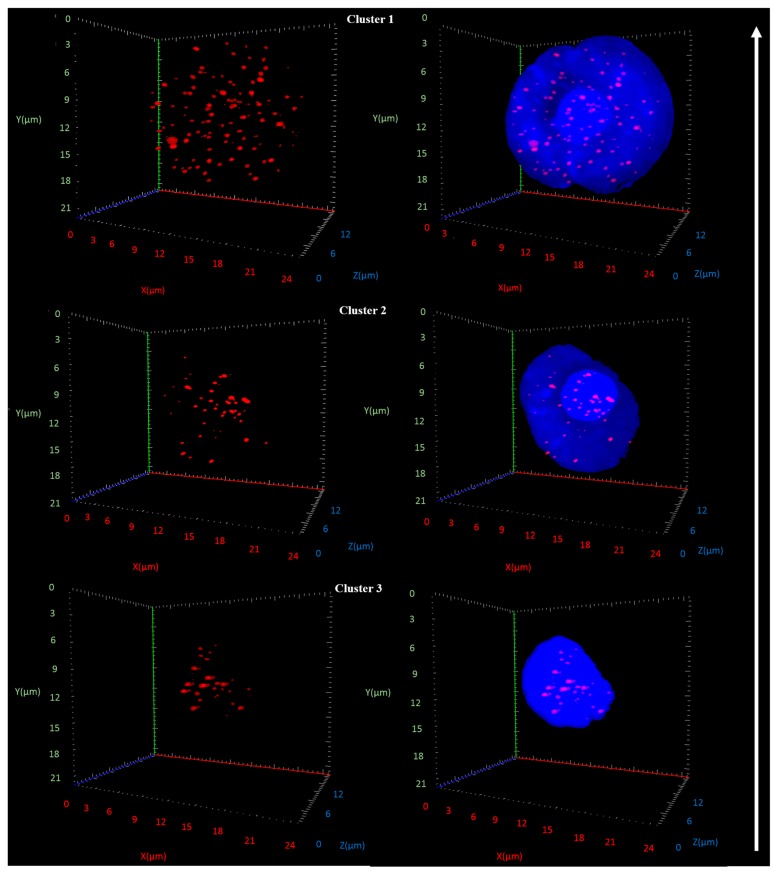
Representative images of CTCs from patients in cluster 1, 2 and 3. Left panels: 3D telomere (red) images. Right panels: 3D telomeres (red) in CTC nuclei (blue). Note the micrometer (μ) scales for nuclear sizes as shown for each 3D image in the *x, y, z* positions of the image. A white arrow indicates the increase in the level of genomic instability from cluster 3 to 2 to 1 (for details, see text).

Figure 2 illustrates 3D images of telomere parameters for a representative patient of each cluster. The representative images show that the organizational and spatial features of telomeres in the nuclei of CTCs increase in their degree of aberration from cluster 3 to 2 to 1. The total number of telomeric signals is highest in cluster 1 as are the number of telomeres per nuclear volume and the nuclear volumes (Figure 2). Cluster 1 shows a representative nucleus with a diameter of 20 μm. Cluster 2 is intermediate between clusters 1 and 3; in this cluster, the numbers of telomeric signals and the numbers of telomeres per nuclear volume are increased compared to cluster 3 but are lower than in cluster 1. Similarly, the nuclear volumes are increased in cluster 2 compared to cluster 3 (nuclear diameters of 13 μm vs. 9 μm, respectively), but below those seen in cluster 1 (Figure 2). Thus, there is a marked increase in the level of aberrant spatial telomere organization from cluster 3 to 2 to 1.

### 2.3. Modeling of 3D Telomeric CTC Parameters

**Figure 3:**
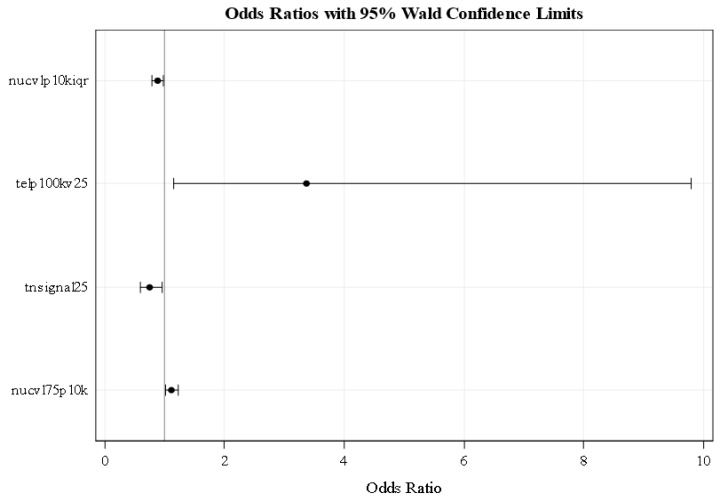
Logistic regression modeling identified four parameters that enable the distinction of stable vs. aggressive disease as measured by 3D telomere profiling in patients with intermediate risk prostate cancer. At a confidence level of 95%, patients with values < 1 are considered to be with stable disease at the time point of the analysis, while patients with values > 1 are considered to be with aggressive disease. The wide 95% confidence interval (CI) observed for telp100kv25 indicates that this predictor is less accurate than the other three that all display a very narrow 95% CI. Abbreviations in this figure: iqr: interquartile range (the difference between the 75th percentile and the 25th percentile); nucvolp10kiqr: nuclear volume (10 × interquartile); telp100kv25: telomeres per nuclear volume (100 × nuclear volume 25th quartile), tnsignal25: total number of telomere signals (25th quartile), nucvol75p10k: nuclear volume (10 × 75th quartile).

We next performed logistic regression modeling using the 3D telomere parameters measured by TeloView [17] (File S1). Logistic regression modeling showed that four parameters separated stable from aggressive disease (Figure 3). At 95% confidence levels, odds ratios of < 1 are indicative of stable disease; odds ratios > 1 are indicative of aggressive disease. The following 3D telomere parameters separated the stable from aggressive disease, nuclear volume (nucvolp10kiqr), telomeres per nuclear volume (telp100kv25), total number of signals (tnsignal25), and nuclear volume (nucvl75p10k).

**Figure 4:**
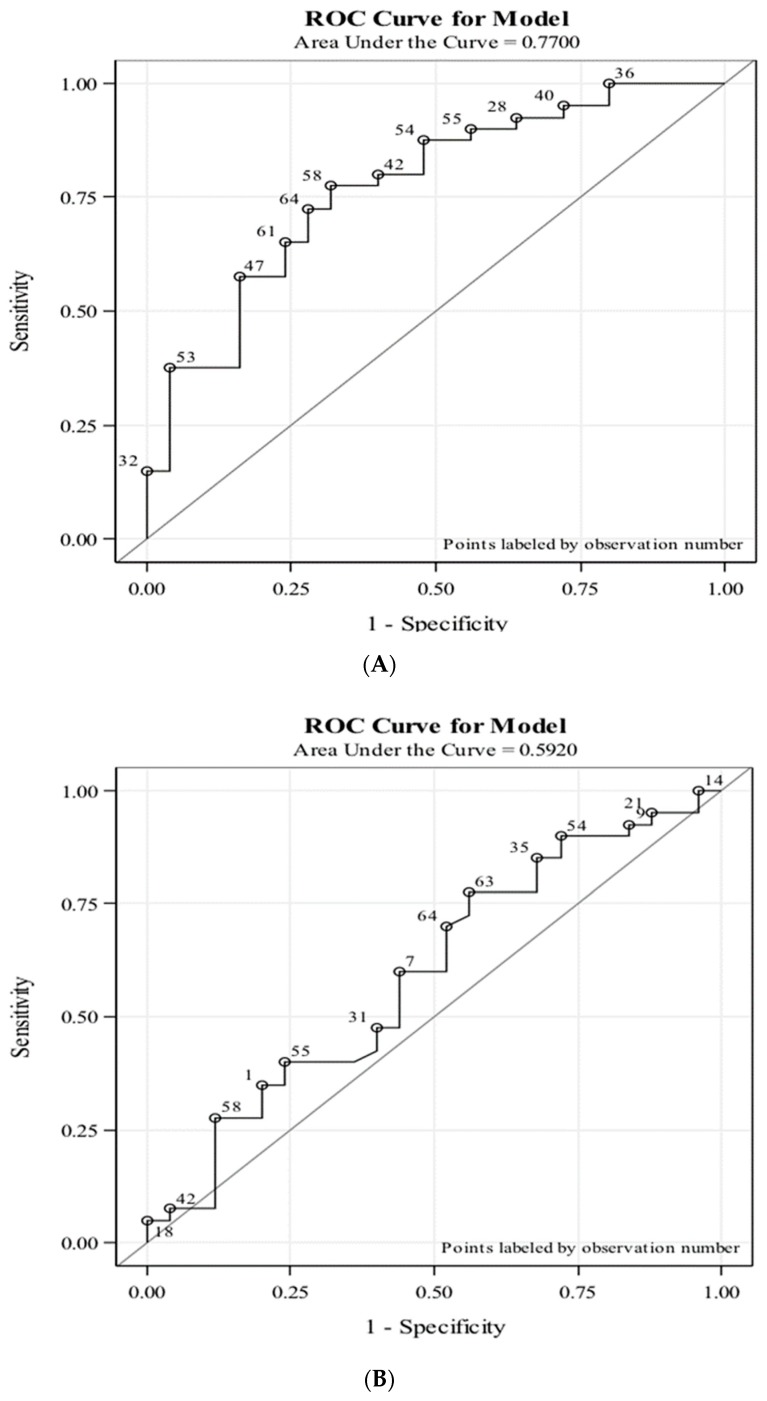
Receiver operating curve (ROC) for 3D telomere analysis of pre-operative CTCs from 65 intermediate risk prostate cancer patients compared to their prostate specific antigen (PSA) values. (**A**): ROC curve for 3D telomere profiling of CTCs. (**B**): ROC curve for PSA. The y axes display the sensitivity and the x axes display the specificity of the assays.

These four predictors were modeled and resulted in a receiver operating characteristic (ROC) curve for this pilot study (Figure 4). For 3D telomere profiling of pre-operative CTCs, the area under the curve (AUC) was 0.77, and a sensitivity of 75% and specificity of 68% were reached (Figure 4A). In contrast, pre-operative PSA of these patients reached an AUC of 0.59 (Figure 4B).

Time to RP varied among the patients included in this study (Section Materials and Methods, Table 1). Patients ≤ 3 months showed the best predicted linear trend, with one degree Mantel–Haenszel chi-sqared (trend test), *p* = 0.008, 75% sensitivity, and 73.33% specificity. Patients ≤ 6 months had better sensitivity (77.14%) but poorer specificity (65.22%). Taken together, the whole group of patients showed a sensitivity of 75% and specificity of 68%. A future and larger cohort study should potentially focus on patients ≤ 3 months of RP, which may further improve predictability.

## 3. Discussion

Intermediate risk prostate cancer represents a challenge to patients and clinicians as this group is heterogenous and the risk to progression unclear. Current PSA screening and biopsies that give Gleason scores (3 + 4 or 4 + 3) do not suffice in predicting disease progression [1,5]. As active surveillance is more readily utilized for low tier intermediate risk patients with 3 + 4 disease, reliability regarding risk stratification is desperately needed. The need for better identification/stratification of indolent or more aggressive disease motivated this study.

To this end, we examined pre-operative CTCs isolated from the blood of 65 intermediate risk prostate cancer patients. 3D telomere profiling was used to assess the level of genomic instability present in the CTCs of each patient. The study was done in a blinded manner. After completion of analysis, final pathology results of RPs became available and patients were identified with “stable” or “upgraded” disease status (Table 1). Forty of the 65 patients were upgraded based on the RP pathology results.

3D telomere profiling as utilized in this report, has previously been applied to other cancers, including neuroblastoma, glioblastoma, myelodysplastic syndromes and acute myeloid leukemia, Hodgkin’s lymphoma, multiple myeloma, and thyroid cancer [8,9,10,11,12,13]. In all cases examined so far, this technology was able to identify patient subgroups.

This current study is, to our knowledge, the first one applying 3D telomere profiling of CTCs to the heterogeneous intermediate risk prostate cancer patients.

Our 3D telomere data relate to the genomic heterogeneity in prostate cancer patients with Gleason 6 and 7 that was reported by Lalonde et al. [1]. Based on genetic profiling, these authors identified four groups of patients with Gleason 6 and 7. Group 4 consisted of patients with so-called quiet genomes due to few genomic alterations. Patients in the latter subgroup had a significantly better prognosis than those in subtypes 1–3. This study also indicated that the four genomic instability-derived subtypes were independent of Gleason score, T category, and prostate PSA in all cohorts. Individual Gleason 6 tumors had a higher percentage of genome alteration than some Gleason 7 (4 + 3) tumors. The percentage of genome alteration was strongly prognostic and independent of clinical covariates. Similarly, Boutros et al. [5] found a high level of heterogeneity in Gleason 7 patients based on copy number variations.

The data obtained with our pilot cohort suggest that 3D telomere profiling is able to stratify intermediate risk prostate cancer patients into risk subgroups. The subgrouping identifies patients with low, intermediate and higher risk of progression as indicated by the centroid cluster analysis of the 3D parameters measured with CTCs. The different levels of genomic instability found in the three clusters correlates with low, medium or elevated risk to progression. Cluster 3 seems to be similar to group 4 in Lalonde’s study, while clusters 2 and 1 appear to correspond to the more aggressive phenotypes of the other subtypes [1]. Of note, our study was based on CTCs, while theirs was based on image-guided biopsies.

In contrast to the cluster analysis that used a combination of 3D telomere parameters, logistic regression modeling identified four 3D telomere parameters that separated patients with aggressive/progressive vs. stable disease at confidence interval (CI) of 95%. ROC curves of 3D telomere profiles obtained from pre-operative CTCs were then created based on these four parameters.

3D telomere profiling in this pilot cohort provided better reads than the ones obtained with 2600 men in a large study focused on PSA levels [35]. In this former study, the AUC was 0.67 [18]. Other studies on PSA found AUCs of 0.55–0.70 (reviewed in Prensner et al. [36]). In the large study reported by Hoffman et al. [35], PSA of 4 ng/ml had a sensitivity of 86% and a specificity of 33%, while PSA of 7 ng/mL had a sensitivity of 32% and a specificity of 56%. 

The area under the curve AUC we obtained using 3D telomere profiling of pre-operative CTCs of 65 men was 0.77. For patients ≤ 3 months, the assay reached 75% sensitivity and 73.33% specificity. Patients ≤ 6 months had better sensitivity (77.14%) but poorer specificity (65.22%). Taking all patients together irrespective of time to surgery, 3D nuclear telomere profiling reached a sensitivity of 75% and specificity of 68%. PSA for our cohort had an AUC of 0.59. Gleason 3 + 4 = 7 vs. 4 + 3 = 7 was unable to predict progressive or stable disease (*p* > 0.6). Thus, similar to previous studies in the same intermediate prostate cancer risk group, PSA and Gleason score are less likely to indicate a patient’s risk to progression than genetic profiling or 3D structural telomere analysis [1,5].

Admittedly, our sample size was small, and therefore future studies should involve a larger cohort and focus on blood and CTC collection from intermediate risk prostate cancer patients ≤ 3 months of RP for the best predictive value to be obtained.

These first data on 3D telomere profiling of intermediate risk prostate cancer patients’ CTCs demonstrate that CTCs contain potentially useful structural genetic information on the risk of prostate cancer progression. This information cannot be gleaned solely from surgical pathology biopsy specimens as important pathological information is not available on original biopsy due to sampling limitations. In our study, 40 of 65 patients were upgraded after RP and needed RP. In contrast, the remaining 25 patients could have been monitored by active surveillance. The stratification of intermediate risk prostate cancer patients into those with indolent or aggressive disease may be predictable based on pre-operative CTC analysis. The information obtained through 3D CTC telomere profiling could have value in pre-treatment discussions surrounding active surveillance and neo-adjuvant and adjuvant therapy provision.

## 4. Materials and Methods

### 4.1. Patients and Collection of Circulating Tumor Cells

Sixty-five treatment-naïve patients with biopsy-confirmed D’Amico-defined intermediate risk prostate cancer consented to the analysis of their CTCs. Time to RP varied among the patients included in this study. Fifty-eight patients (89.23%) were within six months to surgery. Thirty-nine patients (60%) were within three months. Two patients were >eight months prior to RP. The patient characteristics before RP and at RP are summarized in Table 1. Ethics Board approval and informed consent was obtained for the study (HS14085, H2011:336).

A total of 9 mL of blood was collected once per patient pre-RP in Vacutainer^®^ blood collection tubes (with EDTA as an anti-coagulant) and processed within two hours. Patient blood was processed using the ScreenCell^R^ filter method for the separation of prostate CTCs [32]. Briefly, patient blood (3 mL) was pre-cleared with red blood cells lysis buffer (8 mins and 4 mL), and the remaining blood cells were prefixed prior to ScreenCell^®^ (7.50 ± 0.36 μm pore size) filtration to collect cells, which were unable to pass through the filter pores.

### 4.2. Three-Dimensional Telomere Hybridization and Analysis

#### 4.2.1. Quantitative Fluorescent in Situ Hybridization (QFISH)

For QFISH, cells on the filters were incubated in 1 × PBS for 5 min followed by a 10 min fixation in 3.7% formaldehyde/1 × PBS and three washes in 1 × PBS for 5 min each. Filters were treated with 50 μg/mL pepsin (Sigma) in 0.01 M HCl for 10 min at 37 °C, 1 × washed in 1 × PBS for 5 min followed by post-fixation for 10 min in 3.7% formaldehyde/1 × PBS and 3 × washes in 1 × PBS for 5 min each. Filters were dehydrated in an ethanol series (70%, 90%, and 100% ethanol for 3 min each) and air-dried. Cyanine 3 (Cy3)-conjugated telomere peptide nucleic acid (PNA) probe (DAKO, Glostrup, Denmark) was applied (5 µL probe/slide), and following denaturation at 80 °C for 3 min, and hybridization was done for 2 h at 30 °C. Slides were washed in 70% deionized formamide (Sigma, Oakville, ON, Canada) in 10 mM Tris pH 7.4 for 15 min, rinsed in 1 × PBS and followed 1 × by 2 × saline-sodium citrate (SSC) (5 min at 55 °C), 0.1 × SSC, and 2 × SSC/0.05% Tween-20 at room temperature. Filters were again dehydrated in a series of ethanol washes (70%, 90%, 100%) and air-dried. Filters were removed from the metal support ring using an 8mm biopsy punch, placed on a new slide, 4′,6-diamidino-2-phenylindole (DAPI)- stained, mounted with Vectashield (Vector Laboratories, Burlington, ON, Canada), and a coverslip.

#### 4.2.2. Three-Dimensional (3D) Telomere Imaging and Analysis

Slides were imaged on a Zeiss AxioImager Z2 microscope with a Zeiss AxioCam MRmm Rev 3 digital camera using AxioVision Release 4.8.2 (Zeiss, Jena, Germany). A Cy3 filter was used to detect the Cy3 probe nuclear hybridization to telomeric repeats at an exposure time of 546 ms. Exposure times for the DAPI filter differed between slides. Eighty focal planes spaced 200 nm apart were imaged to create three-dimensional nuclear images of the circulating tumor cells and lymphocytes on the filter. Images were deconvolved using a constrained iterative algorithm [37]. For each patient, we examined thirty CTCs and performed 3D telomere hybridizations, 3D quantitative imaging, and analysis of 3D telomere parameters using TeloView^TM^ as described previously [17,25,34]. TeloView^TM^ is proprietary to Telo Genomics (Toronto ON, Canada) and was used with the company’s permission. The program measures telomere signals and their intensities, the presence of telomere clusters (aggregates) that, at 200 nm optical resolution, cannot be further resolved into individual signals, nuclear volumes, and telomeres per nuclear volume. An additional feature of TeloView^TM^ analysis uses the a/c ratio to assess the cell cycle distribution as being G0/G1, S or G2 [17,26]. As the CTCs are collected on filters under mild vacuum, the overall nuclear distribution of telomeres within the oblate spheroid of the nucleus measured through the axes a, b, and c is not identical to their distribution in cells examined in the absence of vacuum. Therefore, the a/c ratio was not determined in the current study as also previously reported [25]. All other telomere parameters were measured.

### 4.3. Statistical Analyses

T-test and generalized linear model (GLM) procedures did not suffice to identify a single predictor stratifying the patients.

Hierarchical centroid cluster analysis identified three clusters of patients. The clusters were formed by combinations of 3D telomere parameters. Nuclear volume (nuclear volume interquartiles (nucvoliqr)), telomeres per nuclear volume (telomeres per nuclear volume, 25th quartile (telpkvol25)), and percentage of great intensities (greatintpct) were best able to stratify patients into three clusters.

Logistical regression modeling assessed the probability of a patient to fall into the “stable” or “aggressive” group. The following parameters remained at the end of the backward stepwise modeling process: nuclear volume (10 × inter quartiles (nucvolp10kiqr)), telomeres per nuclear volume (100 × nuclear volume 25th quartile (telp100kv25)), total number of telomere signals (25th quartile (tnsignal25)), and nuclear volume (10 × 75th quartile (nucvl75p10k)). Adjusted odds ratios with 95% confidence level were estimated. Adjusted odds ratios below 1 were indicative of stable, while odds ratios above 1 were indicative of aggressive disease.

A ROC curve was constructed for the four-parameter telomere model to indicate the concordance between the model and the data. A ROC curve was also constructed for PSA.

## 5. Conclusions

Quantitative 3D telomere profiling allowed for the stratification of the intermediate risk prostate cancer patients into three subgroups, each of which contained patients with different risk to progression. Moreover, logistic regression modeling, identified 3D telomere parameters that separated patients into those with currently stable or progressive disease (with CI of 95%). 3D telomere profiling data therefore suggest that CTCs contain structural genetic information on the risk of progression of intermediate risk prostate cancer patients. This information is not available in original biopsies because of sampling limitations. Thus, a patient’s risk may be predictable based on pre-operative CTC analysis. This information would be highly relevant to pre-treatment discussions surrounding active surveillance and neo-adjuvant and adjuvant therapy provisions.

## Figures and Tables

**Table 1:** Clinical information of 65 intermediate risk prostate cancer patients at biopsy and after radical prostatectomy. Bold highlights patients whose cancer was upgraded after radical prostatectomy (RP). Days to surgery after circulating tumor cells (CTC) collection varied among patients. The results of the hierarchical centroid cluster analysis (Figure 1) are indicated for each patient (clusters 1, 2 or 3).

Patient Number	Gleason	Stage	Prostate Specific Antigen (PSA)	Cluster	Days to Surgery	Gleason Score	Clinical Data
	Before Radical Prostatectomy						After Radical Prostatectomy
1	4 + 3	T2c	8.9	3	1	3 + 4	RP 03/2014 (no tert pattern, pT2c N0), PSA currently undetectable
2	4 + 3	T1c	14.71	1	10	4 + 3	**RP 12/2012 (tert 5 pattern, pT2c N0), PSA currently undetectable**
3	4 + 3	T2b	3.26	1	10	4 + 3	**RP 08/2014 (T3b N1, no tert pattern), PSA currently undetectable**
4	3 + 4	T1c	4.18	1	10	3 + 4	**RP 2/2018 (tert 5 pattern, pT2N0), 9/27/18 PSA < 0.01, PSA currently undetectable**
5	3 + 4	T2a	8.3	2	11	3 + 4	**RP 10/2014 (no tert pattern, pT3b, N1), BCR, RT + ADTx1yr, PSA currently undetectable**
6	3 + 4	T1c	5.7	2	14	3 + 4	RP 08/2012 (no tert pattern, pT2 N0), PSA currently undetectable
7	4 + 3	T1c	3.85	2	15	3 + 4	RP 09/2014 (no tert pattern, pT2c N0), PSA currently undetectable
8	3 + 4	T1c	11.8	3	16	3 + 4	RP 12/2012 (no tert pattern, pT2c N0), PSA currently undetectable
9	3 + 4	T1c	3.5	1	17	3 + 4	**RP 12/2012 (tert 5 pattern, pT3b N0), PSA currently undetectable**
10	3 + 4	T1c	4.93	2	17	3 + 4	**RP 09/2014 (no tert pattern, pT3a N0), PSA currently undetectable, 9/7/17 PSA < 0.01.**
11	3 + 4	T2a	9.5	2	17	3 + 4	RP 09/2014 (no tert pattern, pT2c NX), PSA currently undetectable
12	3 + 4	T2a	5	1	17	3 + 4	**RP 04/2015 (pT3a N0), PSA currently undetectable**
13	4 + 3	T1c	7.86	1	17	4 + 3	**RP 11/2017 (tert 5 pattern, pT2c N0), 1/2/18 PSA < 0.01, PSA currently undetectable**
14	4 + 3 (9/2016), 4 + 3 (11/2017)	T1c	7.22, 7.30	1	17	**4 + 5**	RP scheduled 5/2018, (no tert pattern, pT2 N0), 6/19/18 PSA < 0.01, PSA currently undetectable
15	4 + 3	T2a	5	1	23	4 + 3	**RP 10/2014 (no tert pattern, pT3a, invasion of bladder neck muscle), PSA currently undetectable**
16	3 + 4	T1c	15	1	24	4 + 3	**RP 04/2015 (tertiary 5 pattern, T2c N0), PSA currently undetectable, 11/13/18 PSA < 0.01**
17	4 + 3	T2b	9	1	26	4 + 3	RP 12/2012 (no tert pattern, pT2c N0), PSA currently undetectable
18	4 + 3	T1c	5.56	1	27	4 + 3	**RP June 25, 2018 (tert 5 pattern, T3aN1), 8/20/18 PSA < 0.01**
19	3 + 3 (3/2008), 3 + 3 (2/2009), benign (11/2011), 3 + 3 (3/2014), 4 + 3 (10/2017)	T1c	3.98, 4.97, 5.6, 4.89, 5.71	1	27	4 + 3	**RP June 25, 2018 (tert 5 pattern, T3bN0), 8/8/18 PSA 1.71, 8/28/18 PSA 1.84, ADT Oct 2018 (plan to be on for 6–8 months), 10/9/18 PSA 10.8**
20	4 + 3	T2a	3.8	1	28	3 + 4	RP 09/2014 (no tert pattern, pT2c N0), PSA currently undetectable
21	3 + 4	T1c	5	1	30	3 + 4	**RP 06/2013 (no tert pattern, pT3a N0), PSA currently undetectable**
22	3 + 4	T1c	8.9	1	31	3 + 4	**RP 09/2014 (tert 5 pattern, node + ve), BC failure, received RT /ADT × 2 yrs, PSA currently undetectable**
23	4 + 3	T2c	2.8	3	35	3 + 4	RP 10/2014 (no tert pattern, pT2c N1), PSA currently undetectable, 5/1/18 PSA 0.19, 11/6/18 PSA 0.23
24	4 + 3	T2a	5.02	1	36	4 + 3	RP 01/2014 (no tert pattern, pT2c N0), PSA currently undetectable
25	3 + 4	T1c	27	1	36	3 + 4	**RP 01/2018 (no tert pattern, pT3aN0), 3/7/18 PSA 0.01, PSA currently undetectable**
26	4 + 3	T1c	16	1	37	4 + 3	**RP 04/2014 (tert 5 pattern, pT3b N1), ADT × 2 yrs, PSA currently undetectable**
27	4 + 3	T2a	14.5	1	38	3 + 4	**RP 11/2017 (no tert pattern, p3a N1), 12/18/17 PSA 0.03, 1/30/18 PSA 0.02, PSA currently undetectable**
28	4 + 3	T1c	7.7	1	44	4 + 3	**RP 08/2012, (no tert pattern, pT3a N1) node + ve, salvage ADT 2013, 2/5/16 PSA 0.04**
29	4 + 3	T2a	8.91	2	44	4 + 3	RP Aug 9, 2018 (no tert pattern, T2N0), 10/16/18 PSA 0.01, PSA currently undetectable
30	3 + 4	T1c	11.15	2	45	3 + 4	**RP 08/2013, PSA currently undetectable, (no tert pattern T3a N0 MX), 6/11/18 slow rising PSA 0.24, salvage RT 8/2018**
31	4 + 3	T1c	5.4	1	45	4 + 3	RP 03/2015 (no tert pattern, pT2c N0), PSA currently undetectable
32	3 + 4	T1c	5.88	1	55	3 + 4	**RP 07/2015 (tert 5 pattern, pT2C, N0), PSA currently undetectable**
33	4 + 3	T2a	8.28	1	65	4 + 3	RP June 21, 2018, (no tert pattern, T2N0), 8/28/18 PSA < 0.01
34	3 + 4	T2a	1.23	1	66	3 + 4	RP 03/2014, (no tert pattern, pT2c N0), 11/21/17 PSA 0.01
35	4 + 3	T1c	5.86	1	71	4 + 3	**RP 05/2013 (tert 5 pattern, pT3 N0), BCR, salvage RT 06/2015, PSA currently undetectable**
36	4 + 3	T1c	5.7	1	73	4 + 3	**RP 12/2012 (no tert pattern, pT3a N0), BC failure, salvage RT 09/2014, PSA currently undetectable, BCR 7/31/17 PSA 0.25, 11/6/18 PSA 18.12**
37	3 + 3, 11/2015, 4 + 3 (4/2017)	T1c	9.0, 13.74	1	85	3 + 4	**RP 12/2017 (tert 2 pattern, pT3b N1 MX), 2/27/18 PSA 0.09, ADT 6 months 11/5/18 PSA 0.01**
38	4 + 3	T1c	7.5	1	86	4 + 3	**RP 01/2013 (tert pattern 5, pT2c), PSA currently undetectable, 1/11/18 PSA 0.08**
39	3 + 4	T1c	3.71	1	90	3 + 4	RP April 23, 2018 (no tert pattern, pT2 N0), 6/26/18 PSA 0.01
40	3 + 4	T1c	19.73	1	99	3 + 4	**RP 03/2014 (tert 5 pattern, pT3aN0MX) PSA is currently undetectable, 4/4/18 PSA < 0.01**
41	3 + 4	T2a	2.24	1	101	3 + 4	RP 03/2013 (no tert pattern, pT2c N0, significant volume), PSA currently undetectable
42	3 + 4	T1c	14.51	1	101	3 + 4	**RP 12/2017 (tert 5 pattern, pT3a N0), 3/27/18 PSA 0.12 gradually rising, 10/1/18 PSA 0.25, Adjuvant RT 10/2018**
43	4 + 3	T1c	11.53	2	102	3 + 4	RP 12/2017 (no tert pattern, pT2b N0), 2/22/18 PSA < 0.01, PSA currently undetectable
44	3 + 4, (3/2012), 3 + 4 (1/2013), Benign (9/2014), 4 + 3 (9/2017)	T1c	10.8, 12.64, 8.99, 17.92	1	121	4 + 3	RP 6/1/18 (no tert pattern, pT2N0), 8/17/18 PSA 0.03
45	4 + 3	T2a	9.3	1	128	4 + 4	**RP August 9, 2018. (tert 5 pattern, T3aN1), 10/22/18 PSA 0.77, ADT received 1 dose 11/2018 (plan is for 6–8 months), 11/22/18 PSA 0.69**
46	3 + 4	T2b	3.31	1	134	3 + 4	RP 1/2018, (no tert pattern, pT2 N0), 2/28/18 PSA < 0.01, PSA currently undetectable
47	4 + 3	T1c	5.5	3	138	4 + 3	RP April 23, 2018 (no tert pattern, pT2cN0), 9/4/18 PSA < 0.01, PSA currently undetectable
48	3 + 4	T1c	4.95	1	143	3 + 4	RP 09/2013 (pT2c N0, 75% involvement), PSA currently undetectable, 6/22/18 PSA 0.14
49	4 + 3	T1c	17.08	1	144	3 + 4	RP 6/15/2018 (no tert pattern, pT2N0MX), 9/18/18 PSA < 0.01, PSA currently undetectable
50	4 + 3	T2a	10.34	2	149	4 + 3	**RP 11/2012 (tert pattern 5, pT2c N0), PSA currently undetectable**
51	4 + 3	T1c	6.51	3	156	4 + 3	**RP May 10, 2018, (no tert pattern, pT3aN0), 8/7/18 PSA 0.02**
52	4 + 3	T2a	5.61	1	157	4 + 3	**On AS, progression on F/U biopsy, RP 07/2014 (no tert pattern, pT3a N0), PSA currently undetectable**
53	4 + 3	T1c	7.88	1	162	4 + 3	**RP 7/12/18 (tert 5 pattern, T2N0), 9/26/18 PSA < 0.01**
54	3 + 3 (12/2015), 3 + 3 (1/2017), 4 + 3 (12/2017	T1c	4.25, 12.77, 19.49	1	166		RP Oct 2, 2018 (no tert pattern, T2 N0 MX)
55	4 + 3	T2a	1.41	1	167	4 + 3	**RP 09/2013 (tert 5 pattern, pT3b N1), climbing PSA to 0.14, RT 04/2015, ADT × 2 yrs**
56	3 + 3 (2/2017), 3 + 4 (5/2017)	T1c	18	1	167	4 + 3	**ADT 3 months, RP 3/28/18 (tert 5 pattern, pT2cN0), PSA 0.01**
57	3 + 4	T2a	9.4	2	177	4 + 3	**On AS, grade/volume progression (4 + 3 in 6 cores, Comedo necrosis suggestive of pattern 5), RP 02/2013 (no tert pattern, pT3a N0), PSA currently undetectable**
58.	4 + 3	T1c	12	2	178	3 + 4	**RP Oct 11, 2018 (no tert pattern, T3a N0)**
59	4 + 3 (11/2016), 3 + 4 (1/2018)	T1c	6.68, 7.74	1	184		**RP Nov 22, 2018 (tert 5 pattern, T3b N0)**
60	4 + 3	T1c	11.13	1	204	4 + 3	**RP 8/23/2018 (tert 5 pattern, T3a N0), 9/24/18 PSA 0.01**
61	3 + 4	T2b	6.7	1	207	3 + 4	RP Nov 5, 2018 (no tert pattern T2 N0)
62	3 + 4	T1c	8.48	1	209	4 + 5	**RP Nov 20, 2018 (tert 3 pattern, T2N0MX)**
63	3 + 4	T1c	9	2	225	3 + 4	RP 04/2013 (no tert pattern, pT2c N0), PSA currently undetectable
64	4 + 3	T1c	16.55	1	282	4 + 3	**RP Oct 19, 2018 (no tert pattern, T3b N1)**
65	3 + 4	T2	1.84	1	317	3 + 4	**RP 11/29/18 (no tert pattern, T3a N0)**

## Supplementary Materials

The following are available online at https://www.mdpi.com/2072-6694/11/6/855/s1, File S1: TeloView results from each of the 65 patients.
